# Fracture of a femoral hemodialysis catheter during placement in a man with metastatic cancer

**DOI:** 10.1002/ccr3.1372

**Published:** 2018-01-26

**Authors:** Xiang‐Yang Li, Hai‐Yan He, Pearl Pai

**Affiliations:** ^1^ Department of Nephrology University of Hong Kong‐Shenzhen Hospital Shenzhen China; ^2^ Department of Medicine Queen Marry Hospital University of Hong Kong Hong Kong China

**Keywords:** Catheter, dysfunction, fracture, hemodialysis

## Abstract

Central venous catheterization can be challenging in patients that had undergone repeated catheter placements. Ultrasound scan may overlook venous stenosis which is better visualized using venography. The use of venography should be considered to assess for venous stenosis or vascular anomalies in individuals with multiple catheterizations or in close proximity to cancer.

## Clinical Case

This case report describes the fracture of a temporary femoral hemodialysis catheter during placement in an old man with gastric carcinoma and extensive abdominal and retroperitoneal metastasis, in an attempt to draw lessons from and nurture experience among practitioners.

A 76‐year‐old man was admitted with acute kidney injury and anuria. He underwent a subtotal gastrectomy for gastric carcinoma 7 years ago. Three months prior, he had a similar admission with anuria which required temporary hemodialysis. At that time, a computed tomography and magnetic resonance imaging of his abdomen revealed an atrophic right kidney, extensive intra‐abdominal metastasis, and retroperitoneal involvement causing bilateral hydronephrosis. A temporary double lumen dialysis catheter was inserted in the right femoral vein for acute hemodialysis. A double J stent was placed in the left ureter. His urine volume gradually returned. The dialysis catheter was removed after 7 days. During his second admission with acute oliguric kidney injury, ultrasonography of his bilateral femoral and iliac veins revealed no stenosis or thrombus in femoral and distal iliac veins. A 19.5 cm/13.5 Fr acute dual lumen dialysis catheter [Mahurkar, Covidien, Minneapolis, MN] was placed into right femoral venous using universal Seldinger technique. The procedure was relatively straightforward although the patient experienced some moderate, transient pain during the advancement of the catheter. At the end of the procedure, there was no flow in one of the two lumens.

**Figure 1:**
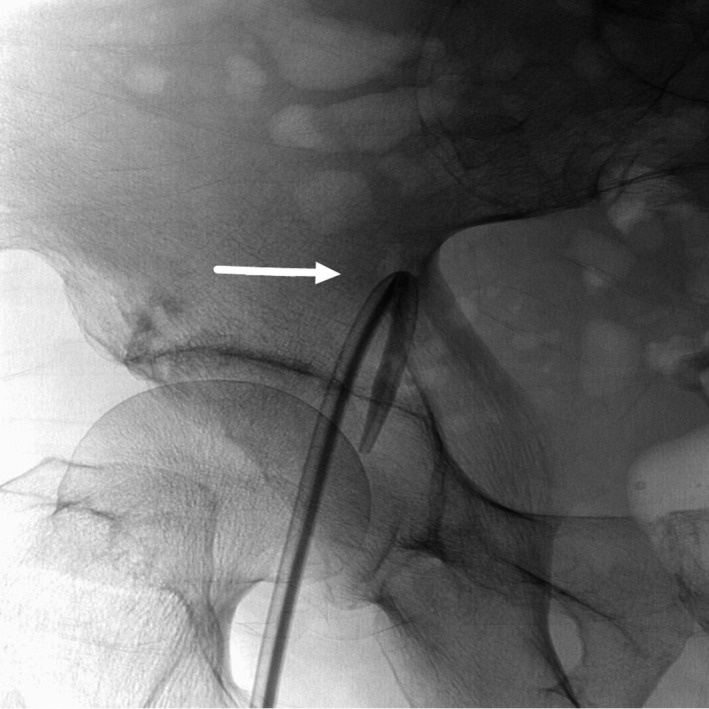
Radiography showing the curved and fractured catheter in the right femoral and iliac veins (arrow).

**Figure 2:**
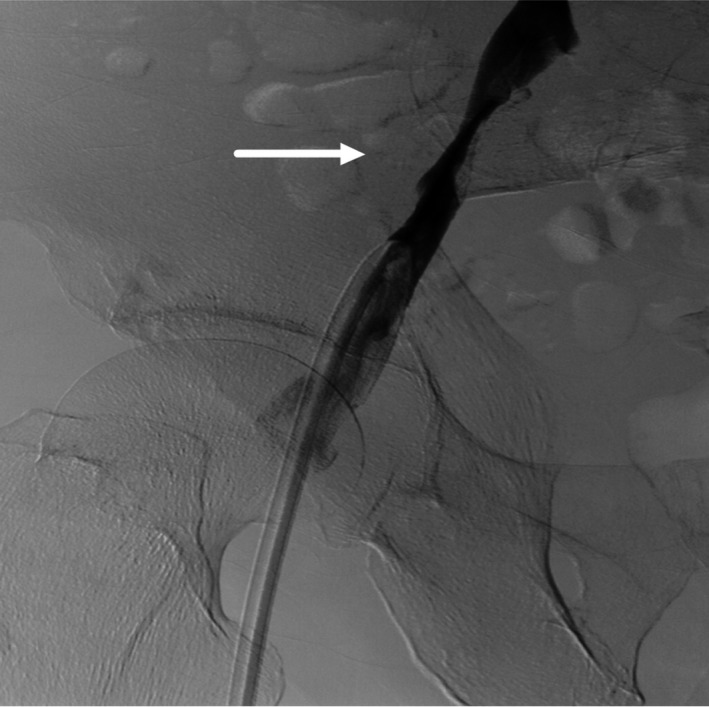
Venography found a stenosis in the middle of right iliac vein (arrow).

Subsequent radiography showed that the end of the catheter had doubled back and the first 4 cm of the catheter had fractured (Fig. 1). Venography showed a stenosis in the middle of his right iliac vein (Fig. 2). The stenosis was thought to have veered the curve end of the guide wire back toward the direction of advancement, and “misguided” the catheter which subsequently broke off. A second dialysis catheter was inserted successfully in the right internal jugular vein under screening. The fractured catheter tip was removed surgically under direct vision. The blood vessel wall was incised over the lodged fragment, the fractured tip was removed, and the incision closed. The narrowing of the right iliac vein was thought to be due to previous femoral catheter placement and associated thrombus formation, or possibly metastatic tumor invasion of the blood vessel wall. More attention and vigilance are required in future similar cases.

## Authorship

XY‐L: conceived the study and did the writing‐up. HY‐H: acquired the data. P‐P: supervised and provided critical revision of the article.

## Conflict of Interest

The authors have declared that no conflict of interest exists.

